# The MEK Inhibitors Trametinib and Cobimetinib Induce a Type I Interferon Response in Human Keratinocytes

**DOI:** 10.3390/ijms18102227

**Published:** 2017-10-24

**Authors:** Daniela Lulli, Maria Luigia Carbone, Saveria Pastore

**Affiliations:** Laboratory of Experimental Immunology, Istituto Dermopatico dell’Immacolata, IRCCS, 00167 Rome, Italy; d.lulli@idi.it (D.L.); marialuigia.carbone@idi.it (M.L.C.)

**Keywords:** dabrafenib, vemurafenib, signal transduction, interferon κ, chemokine

## Abstract

Mitogen-activated protein kinase kinases (MEK) 1 and 2 have crucial roles in tumorigenesis, cell proliferation, and protection from apoptosis, and their inhibition is therefore an attractive therapeutic strategy in cancer. Orally available and highly selective MEK inhibitors have been developed and assessed in numerous clinical trials, either alone or in combination with cytotoxic chemotherapy and/or other targeted agents. Of note, a complex picture of class-specific adverse effects associates with these drugs, frequently including inflammatory skin rash. Here, we investigated the response of normal human keratinocytes to the MEK inhibitors trametinib and cobimetinib, alone and in combination with the v-Raf murine sarcoma viral oncogene homolog B (BRAF) inhibitors dabrafenib and vemurafenib, in terms of signal transduction and de novo gene expression. MEK inhibitors triggered enhanced expression of interferon regulatory factor 1 (IRF1) and phosphorylation of signal transducer and activator of transcription 1 (STAT1), and up-regulated the keratinocyte-specific type I interferon κ (IFN-κ), the anti-viral effectors interferon-induced tetratricopeptide repeats (IFIT) 1 and 2, and the pro-inflammatory chemokine (C-C motif) ligand 2 (CCL2) and the C-X-C motif chemokine 10 (CXCL10), both at the mRNA and protein level. Impairment of IRF1 expression, or abrogation of STAT1 phosphorylation due to *IFN-κ* gene silencing, suppressed anti-viral and pro-inflammatory gene expression. These data suggest that, similar to what we observed for epidermal growth factor receptor (EGFR) blockade, MEK inhibition activates a type I interferon response, which is now recognized as an effective anti-cancer response, in human epidermal keratinocytes.

## 1. Introduction

Many human cancers contain activating mutations in genes encoding the fundamental signaling cascade constituted of receptor tyrosine kinases, including epidermal growth factor receptor (*EGFR*), small GTPase Ras (*RAS*), v-Raf murine sarcoma viral oncogene homolog B (*BRAF*), Raf-1 proto-oncogene (*CRAF*), and the mitogen-activated protein kinase kinases (*MEK*) *1* and/or *2* [1]. These driving oncogenes lead to enhanced dependency on extracellular signal-regulated kinases (ERK) 1 and ERK2 (ERK) signaling and to amplification of ERK-driven cellular processes that are also cancer hallmarks, including sustained proliferation, resistance to cell death, activation of invasion, and metastasis [2]. In this context, the identification of activating *BRAF* mutations (most notably, *BRAF-V600E*) in a large number of human tumors led to the development of *BRAF-V600E* selective inhibitors and the regulatory approval of vemurafenib and dabrafenib [1]. Quite unexpectedly, these BRAF inhibitors were shown to possess the paradoxical effect of activating RAF and downstream ERK in cells with wild-type *BRAF* including skin keratinocytes, with consequent high incidence of hyper-proliferative disorders, including squamous cell carcinoma, in patients treated with these drugs [3,4,5]. To oppose this peculiar toxicity and improve therapeutic efficacy, the development of MEK inhibitors became a priority. MEK1 and MEK2 are dual-specificity kinases that catalyze activating phosphorylation at both tyrosine and threonine residues in ERK, in its turn the only known physiological substrate of MEK [6]. Thus, MEK is a potential bottleneck in the activation of diverse cellular responses of key importance for tumorigenesis [7]. Since the discovery of the first ERK experimental inhibitor PD98059 20 years ago [8], new MEK inhibitors occupying highly specific allosteric sites of the target molecules have provided the opportunity to achieve higher selectivity and have also contributed to validate MEK as a cancer drug target [9]. Two allosteric MEK inhibitors have received regulatory approval for the treatment of human cancers, namely trametinib and cobimetinib, in a protein kinase-specific drug discovery that continues today [10]. Finally, therapies that associate BRAF inhibitors with MEK inhibitors (dabrafenib with trametinib and vemurafenib with cobimetinib) are the current treatment strategies for *BRAF*-mutant advanced cancers, including melanoma [11,12].

The therapeutic use of kinase inhibitors and biological agents targeting kinase signaling pathways has introduced new toxicities in oncology clinics [5]. In particular, treatments that include EGFR inhibitors or MEK inhibitors typically associate with an increased risk of all-grade inflammatory skin rash characterized by papulo-pustular eruptions and acneiform dermatitis [13,14]. In our search for the molecular mechanisms underlying the pro-inflammatory and cytotoxic potential of EGFR inhibitors in the skin, we recently documented drug-induced activation of a type I interferon (IFN) innate immune response, with enhanced expression of the keratinocyte-specific IFN-κ, a number of anti-viral effectors including distinct members of the interferon-induced tetratricopeptide repeats (IFIT) family and immune mediators [15]. The purpose of the present work was to verify that the newly introduced MEK inhibitors share this type of bioactivity in normal human keratinocytes. Our results confirmed this hypothesis, and hence allow us to include these agents among the drugs that potentially contribute a type I IFN activation in the skin. Of major relevance, a growing body of literature indicates that the success of a variety of anti-cancer treatments implicates the induction of type I IFN signaling and shows that intra- and peri-tumoral expression levels of type I IFNs and IFN-stimulated genes correlate with favorable disease outcome in several cohorts of cancer patients [16,17,18]. Hence, beyond its plausible role in the elicitation of undesired skin-specific side effects, activation of type I IFN might be considered one of the anti-cancer mechanisms triggered by MEK inhibitors.

## 2. Results

### 2.1. Trametinib and Cobimetinib Induce a Type I Interferon Response in Keratinocytes

**Figure 1:**
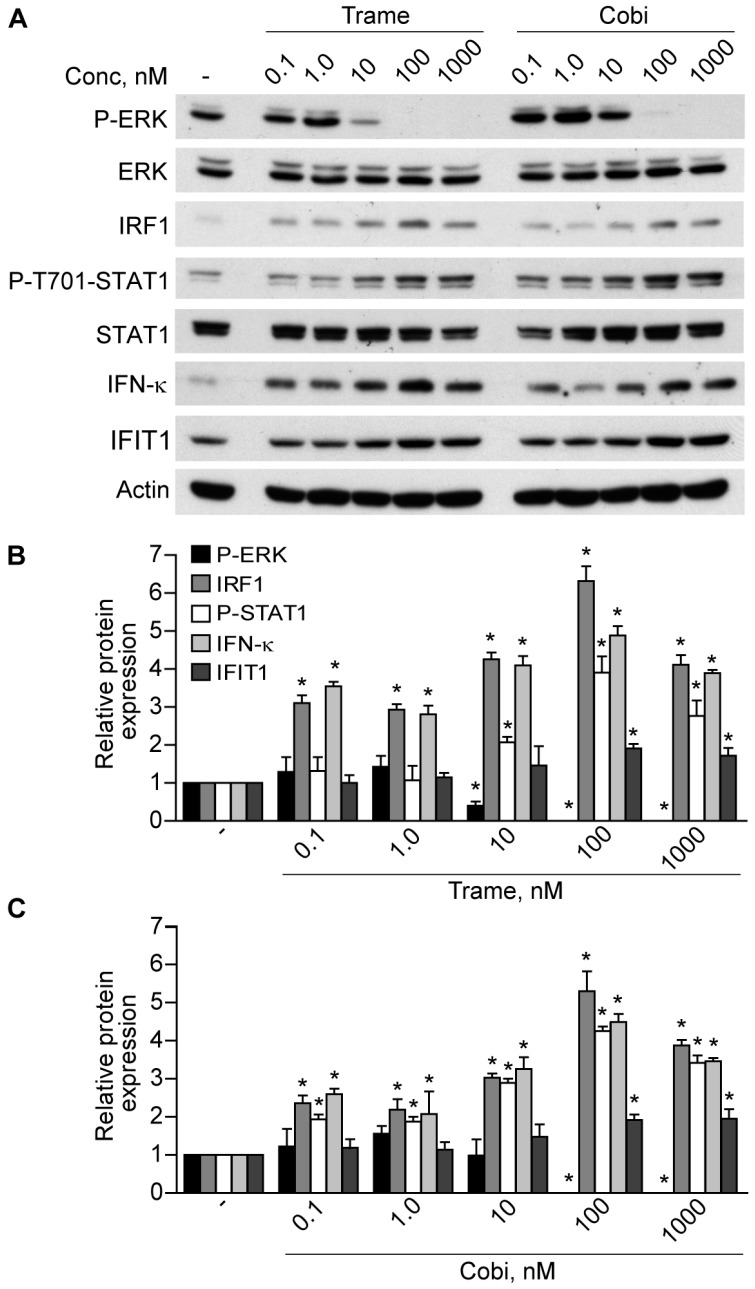
Trametinib and cobimetinib activate interferon regulatory (IRF1) and signal transducer and activator of transcription 1 (STAT1) dose-dependently. (**A**) Western blot analysis of total-cell extracts of normal human keratinocytes treated for 6 h with escalating doses of trametinib (Trame) and cobimetinib (Cobi). The protein species were detected at the expected molecular weights *per* their datasheets, as follows: extracellular signal-regulated kinases (ERK) 1/2), 44 and 42 kDa; IRF1, 48 kDa; STAT1 (STAT1, α/β isoforms), 91 and 84 kDa; actin, 43 kDa; interferon κ (IFN-κ), 26 kDa; interferon-induced tetratricopeptide repeats 1 ( IFIT1), 56 kDa; (**B**,**C**) protein expression was analyzed in cells from different healthy donors (*n* = 4). The ratios of phospho-ERK (P-ERK), IRF1, phospho-Tyr701-STAT1 (P-T701-STAT1), IFN-κ, and IFIT1 to their loading control (actin) were calculated and compared to the untreated condition (−). * *p <* 0.05. Data are expressed as the mean ± standard deviation (S.D.).

Previously, we reported that EGFR inhibitors and the prototypic MEK inhibitor PD98059 led to activation of the type I interferon response in human keratinocytes, with up-regulation of numerous anti-viral effectors and a small group of genes specifically involved in immune cell-cell signaling, including the chemokine (C-C motif) ligand 2 (CCL2) and the C-X-C motif chemokine 10 (CXCL10) [15]. Our data emphasized the pro-apoptotic and pro-inflammatory potential of drugs targeting the EGFR-ERK axis in the epidermis, with implications for their toxicity but also for activation of the anti-cancer defense in the skin. Recently, two highly selective MEK inhibitors, namely trametinib (Trame) and cobimetinib (Cobi), have been introduced, either alone or in combination with BRAF inhibitors, in the treatment of advanced cancers, including metastatic melanoma [11]. We asked whether these new anti-MEK drugs could also trigger a type I interferon response. Here we show that, similar to what we described for EGFR inhibitors, both Trame and Cobi did induce significant interferon regulatory factor 1 (IRF1) up-regulation and signal transducer and activator of transcription 1 (STAT1) phosphorylation concomitant with progressive, dose-dependent inhibition of ERK phosphorylation (Figure 1). These signal transduction events were paralleled by significantly enhanced cell-associated levels of the keratinocyte-specific type I IFN-κ at all drug doses (Figure 1B,C), and also of the anti-viral effector IFIT1, as observed at 100 and 1000 nM concentration (Figure 1B,C).

**Figure 2:**
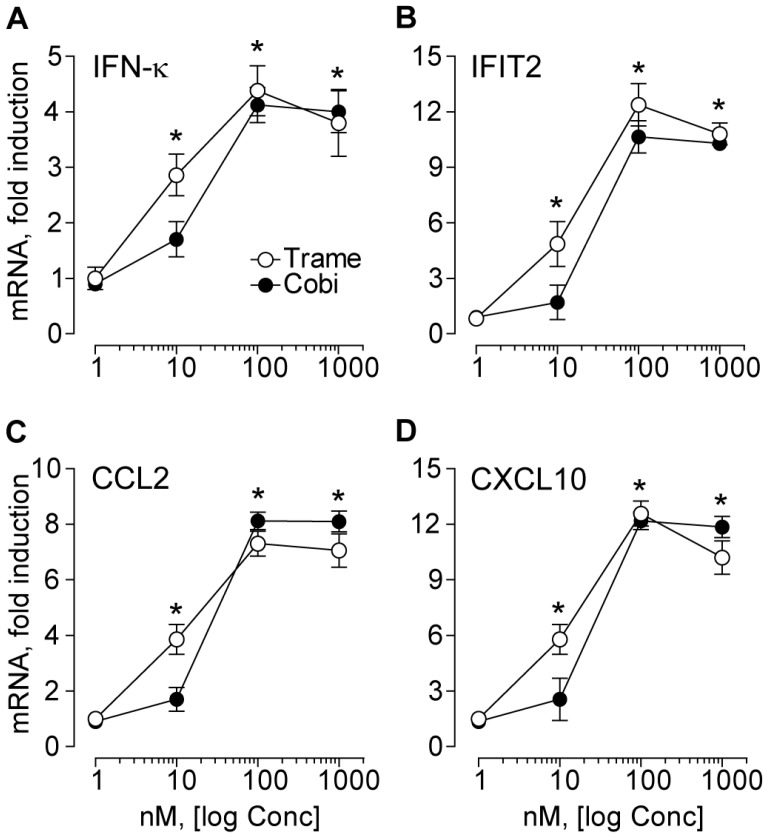
Trametinib and cobimetinib induce de novo gene expression dose-dependently. (**A**–**D**) quantitative real-time RT-PCR analysis was performed in total RNA extracted from normal human keratinocytes treated for 6 h with the two distinct drugs, at the concentrations indicated in the figure. Data are expressed as mean ± S.D. (*n* = 4 per condition). * *p* < 0.05 versus untreated controls.

Gene expression analysis confirmed dose-dependent up-regulation of *IFN-κ* transcript, which was statistically significant at 10 nM compared to untreated controls (*p* < 0.05), and maximal at 100 nM Trame or Cobi (Figure 2A). A similar profile was found for the transcripts of the anti-viral pro-apoptotic effector *IFIT2* (Figure 2B) and of the chemokines *CCL2* (Figure 2C) and *CXCL10* (Figure 2D). Hence, both these MEK inhibitors activated a gene expression program that closely reproduced what we previously observed with EGFR inhibitors or PD98059 in human keratinocytes [15].

### 2.2. Activation of Type I Interferon Signature is Maintained When Mitogen Activated Kinase Kinase (MEK) Inhibitors Are Associated to v-Raf Murine Sarcoma Viral Oncogene Homolog B (BRAF) Inhibitors

**Figure 3:**
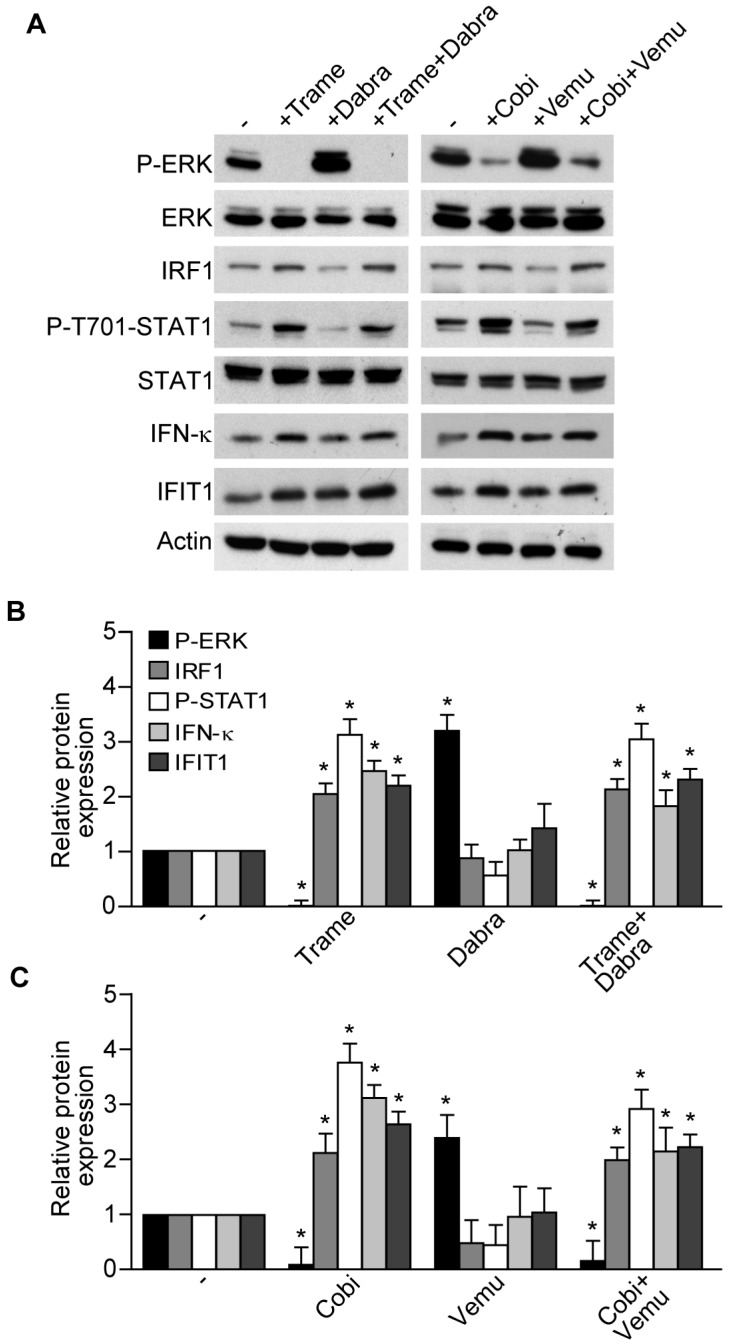
Activation of type I interferon signature is maintained when MEK inhibitors are associated to BRAF inhibitors. (**A**) Western blot analysis of total-cell extracts of normal human keratinocytes treated for 6 h with the MEK inhibitors Trame and Cobi (both at 100 nM), or the BRAF inhibitors dabrafenib (Dabra, 100 nM) and vemurafenib (Vemu, 1 μM), either alone or in combinations; (**B**,**C**) the ratios of protein expression of P-ERK, IRF1, P-T701-STAT1, IFN-κ, IFIT1 to their loading control (actin) were confronted with the no-treatment group (−). * *p* < 0.05. The results are representative of experiments on cells from four distinct donors. Data are expressed as the mean ± S.D.

At present, the combinations of the BRAF inhibitors dabrafenib (Dabra) and vemurafenib (Vemu) with the MEK inhibitors, Trame and Cobi, respectively, have become a new standard of care for the treatment of *BRAF*-mutant metastatic melanoma due to their improved efficacy and reduced toxicity as compared to BRAF inhibitors alone [11,12]. In particular, BRAF inhibitors are responsible of a paradoxical ERK activation in non-*BRAF*-mutant cells including skin keratinocytes, leading to their hyper-proliferation and high incidence of epidermal neoplasms [5]. Notably, this peculiar skin toxicity is efficiently overcome by their association with MEK inhibitors [11,12]. In keeping with this evidence, we found that Dabra and Vemu alone significantly enhanced ERK phosphorylation compared to basal levels, whereas their combinations with Trame and Cobi were characterized by suppression of ERK phosphorylation, concomitant with significantly enhanced IRF1 and phosphorylated STAT1 (Figure 3A–C).

**Figure 4:**
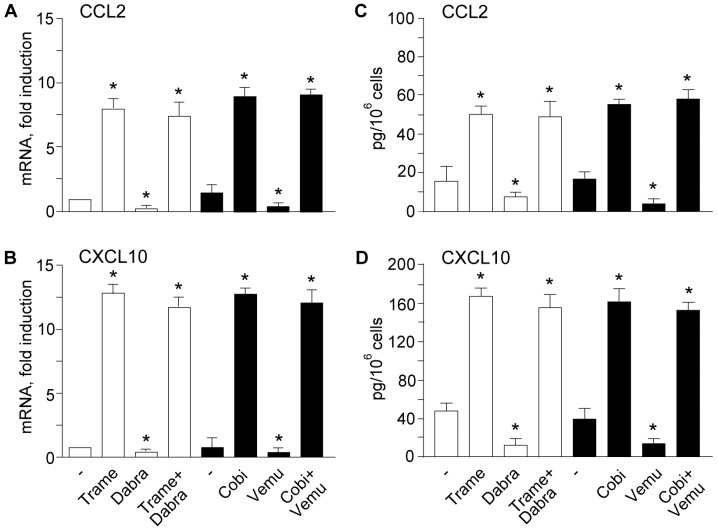
Enhanced expression of the pro-inflammatory chemokine (C-C motif) ligand 2 (CCL2) and the C-X-C motif chemokine 10 (CXCL10) is conserved when MEK inhibitors are associated to BRAF inhibitors. Quantitative real-time RT-PCR analysis of *CCL2* (**A**) and *CXCL10* (**B**) transcripts was performed in total RNA extracted from keratinocytes treated for 6 h with the MEK inhibitors Trame and Cobi (both at 100 nM) or the BRAF inhibitors Dabra (100 nM) and Vemu (1 μM), either alone or in the respective combinations. Protein quantification by ELISA of CCL2 (**C**) and CXCL10 (**D**) was performed on culture supernatants treated for 24 h. * *p* < 0.05 versus untreated controls (−). Data are representative of four independent experiments. Data are expressed as the mean ± S.D.

Up-regulation of IFN-κ and IFIT1 protein levels (Figure 3A–C), and of the chemokines CCL2 and CXCL10, both as transcripts (Figure 4A,B) and proteins released in cell supernatants (Figure 4C,D) confirmed the persistence of MEK inhibitor-associated type I IFN signature in these drug associations.

### 2.3. Impact of Interferon Regulatory Factor 1 (IRF1) and Activated Signal Transducer and Activator of Transcription 1 (STAT1) in MEK Inhibitor-Induced Gene Expression

**Figure 5:**
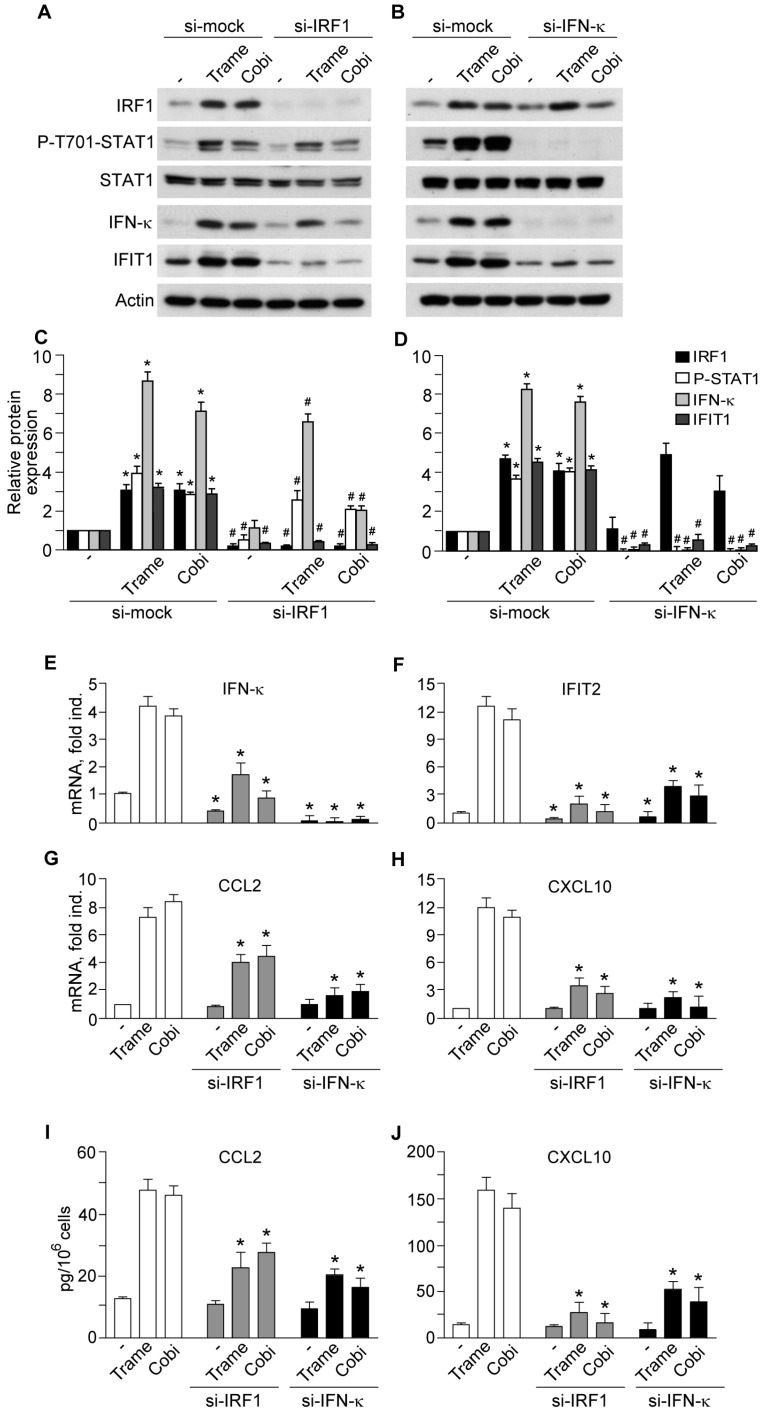
Activation of IRF1 and STAT1 is implicated in the enhanced expression of anti-viral effectors IFIT1 and 2, and the chemokines CCL2 and CXCL10 induced by MEK inhibitors. (**A**,**B**) Western blot analysis of total cell lysates of keratinocytes treated for 6 h with the MEK inhibitors Trame and Cobi (both at 100 nM) were performed after 48 h transfection with irrelevant small interference RNA (si-mock) and specific *IRF1*-targeted si-RNA (*si-IRF1*), or *IFN-κ*-targeted si-RNA (*si-IFN-κ*); (**C**,**D**) the ratios of protein expression of IRF1, P-T701-STAT1, IFN-κ and IFIT1 to their loading control (actin) were calculated. * *p* < 0.05 versus untreated controls (−); #, *p* < 0.05 versus same treatment (untreated controls, Trame or Cobi) in si-mock control cultures; (**E**–**H**) quantitative real-time RT-PCR analysis was performed in total RNA extracted from normal human keratinocytes treated for 6 h with the two distinct drugs. * *p* < 0.05 versus same treatment (untreated control, Trame, or Cobi) in control cultures; (**I**,**J**) protein quantification by ELISA of CCL2 and CXCL10 was performed on keratinocytes supernatants at 24 h. * *p* < 0.05 versus same treatment (untreated control, Trame, or Cobi) in control cultures. Data are indicative of experiments on cells from four different healthy donors. Data are expressed as the mean ± S.D.

Small interference RNA designed to specifically target *IRF1* (*si-IRF1*) significantly impaired Trame- or Cobi-dependent STAT1 phosphorylation, as well as basal and drug-induced expression of IFN-κ (Figure 5A,C,E), IFIT1 protein (Figure 5A,C), and, analogously, *IFIT2* transcript (Figure 5F). Moreover, *si-IRF1* significantly suppressed MEK inhibitor-induced CCL2 (Figure 5G,I) and CXCL10 (Figure 5H,J), both at transcript and released protein levels. By contrast, *IFN-κ* silencing (*si-IFN-κ*) abrogated STAT1 phosphorylation but did not affect IRF1 relevantly (Figure 5B,D), and was associated to prominent reduction of IFIT1 protein (Figure 5B,D) and *IFIT2* transcript (Figure 5F), as well as of both transcript and protein level of CCL2 (Figure 5G,I) and CXCL10 (Figure 5H,J). These data confirmed our previous results documenting the functional involvement of IRF1 and STAT1 in the enhanced expression of anti-viral and pro-inflammatory genes due to EGFR-ERK inhibition in human keratinocytes [15].

## 3. Discussion

**Figure 6:**
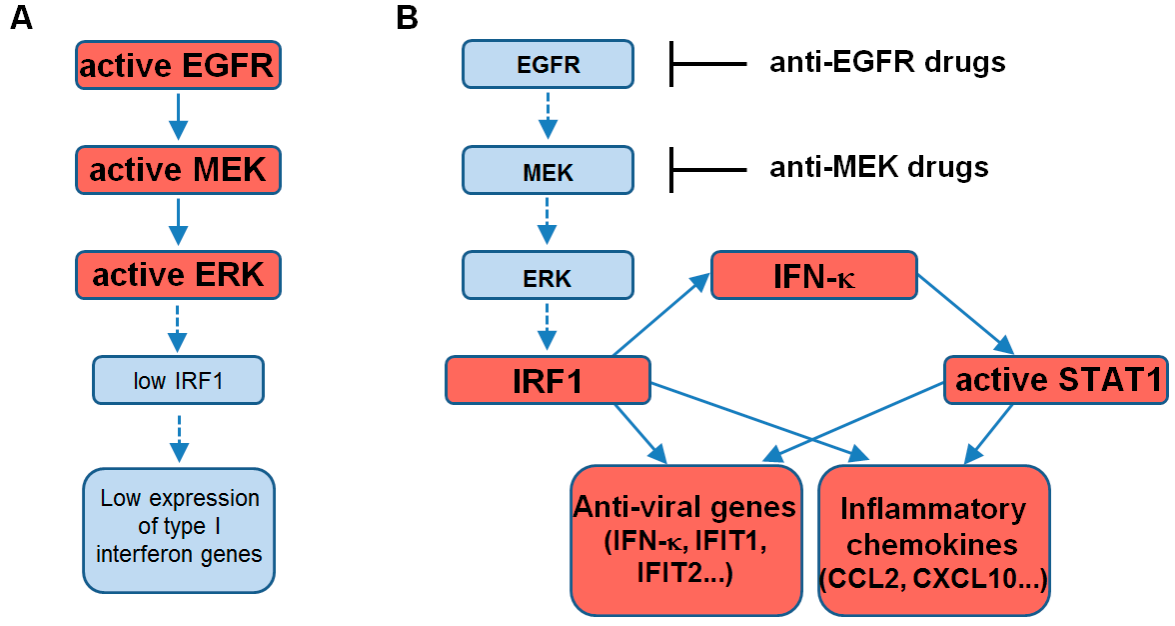
Schematic representation of the impact of EGFR-ERK pathway on the type I interferon response in human keratinocytes. (**A**) Through a molecular mechanism not yet detailed, active EGFR-ERK pathway down-regulates the transcription factor IRF1, thereby keeping under control the expression of genes responsible of the type I interferon response; (**B**) Impairment of the EGFR-ERK pathway due to anti-EGFR and anti-MEK drugs leads to enhanced levels of IRF1. This transcription factor is actively implicated in the expression of the type I interferon gene cluster, including *IFN-κ*, in its turn responsible of STAT1 activation and eventual up-regulation of genes involved in the anti-viral defense and in the immune response. Dashed arrows indicate suppressed steps in the signaling pathway. T bars indicate drug-mediated inhibition.

Here, we verified that the highly selective MEK inhibitors trametinib and cobimetinib, either alone or in combination with BRAF inhibitors, are able to mount a type I IFN response in normal human keratinocytes. In a whole-genome gene expression search for the molecular mechanisms underlying the persistent skin inflammation caused by the EGFR inhibitors, we recently found that these cells responded to these drugs with enhanced expression of numerous anti-viral genes, including the keratinocyte-specific *IFN-κ*, *IFIT1*, and *IFIT2*, and a limited number of key pro-inflammatory mediators, including the T cell, monocyte and dendritic cell chemoattractant *CCL2* and the T cell-selective chemokine *CXCL10* [15]. The present report extends these findings to the MEK inhibitors trametinib and cobimetinib. The transcription factor IRF1 confirmed its primary involvement in this response, which was dramatically impaired by its silencing. Notably, IRF1 binding sites can be found also in *STAT1* promoter [19], thus explaining our observation of a reduction of STAT1 levels in the *si-IRF1* condition. Enhanced expression and/or post-translational activation of IRF1 concomitant to MEK/ERK inhibition has been described by numerous independent groups as previously commented [15], although the underlying molecular mechanisms remain partially defined [20]. Due to its central role in a number of vital cell processes, the ERK pathway is fine-tuned by a complex network of negative feedback controls, mainly dependent on ERK’s phosphorylation/dephosphorylation state [21]. Pharmacological abrogation of ERK activity leads to collapse of these regulatory mechanisms and ERK reactivation, as observed in cancer cells [9]. Hence, it is reasonable to hypothesize that the ERK functional state is involved in a counter-balance relation with a number of other kinases/phosphatases, including critical transducers of the type I IFN signaling cascade. Also, *IFN-κ*-selective gene silencing, with consequent complete abrogation of STAT1 phosphorylation, led to suppression of the type I IFN response triggered by MEK inhibitors. These results emphasize the critical role of IRF1 and STAT1 in the enhanced expression of distinct anti-viral effectors, including IFIT1 and IFIT2 [22], and pro-inflammatory mediators characterizing the type I IFN response (Figure 6).

Hence, commonalities in the clinical presentation of skin-directed adverse effects due to the distinct classes of anti-EGFR and anti-MEK inhibitors [5,23] could rely on the pharmacologically induced, improper activation of the innate antiviral host defense in the epidermis.

The family of the type I *IFNs* consists of genes encoding multiple IFN-α subtypes, one IFN-β, and a number of less-studied subtypes that include the keratinocyte-specific IFN-κ. All type I IFNs bind a ubiquitously expressed heterodimer receptor, whose ligation causes recruitment and phosphorylation of STAT1 and STAT2. In particular, phosphorylated STAT1 can homo-dimerize or hetero-trimerize with STAT2 and IRF9, these last both highly expressed proteins in keratinocytes [15], and eventually transactivate all STAT1-dependent promoter elements, including the chemokines *CCL2* and *CXCL10* [24]. Notably, the pro-inflammatory impact of type I interferons on the skin is well documented. Infiltrating plasmacytoid dendritic cells, the richest source of IFN-α in the whole organism, can be found in the early lesions of psoriasis, and the release of IFN-α is implicated in the elicitation of this chronic, relapsing inflammatory disease [25]. Subcutaneous injections of IFN-β trigger an inflammatory skin reaction associated to enhanced CCL2 and CXCL10 [26]. A consistent body of evidence also indicates that over-activation of the type I IFN response is a characteristic feature of numerous skin-directed immune-mediated and autoimmune diseases [23,27]. In a chronic model of systemic scleroderma-like graft-versus-host disease, inflammation and vascular damage was shown to depend specifically on the type I IFN signaling generated in the skin, not in infiltrating immune cells [28]. Furthermore, increased production of IFN-κ by epidermal keratinocytes was recently proposed as a major pathogenic driver in cutaneous lupus erythematosus [29]. Elevated expression of CXCL10 by skin keratinocytes and consequent massive infiltration by CXCR3+ CD8+ lymphocytes were previously associated to cutaneous lupus erythematosus lesions [30]. Of major relevance, spontaneous regression of melanoma and other melanocytic lesions were associated with activation of endogenous type I interferons and infiltration by plasmacytoid dendritic cells and CXCR3+ cytotoxic T cells, these last recruited by elevation of CXCL10 [31]. Enhanced expression of the inflammatory chemokine CCL2 could also play a protective role against the tumor through the recruitment of type 1 cytotoxic γδ T lymphocytes to tumor beds [32,33]. High number of tumor infiltrating lymphocytes correlate with increased expression of a cluster of chemokines that comprise CCL2 and CXCL10 [34], with CXCL9 and CXCL10 critically involved in the recruitment of effector CD8+ T cells [35].

Type I IFNs are now recognized as effective anti-cancer agents through pleiotropic effects on multiple cell types [18,36]. It is now clarified that type I IFNs exert direct anticancer effects by activating interferon α/β receptor signaling in malignant cells, thereby inhibiting cell cycle progression, promoting terminal differentiation, and inducing apoptosis. In addition, spontaneous innate immune sensing of cancers leading to adaptive immune responses is dependent on host type I IFN production [17,37]. Indeed, the success of conventional chemotherapeutics, epigenetic drugs, targeted anti-cancer therapy, and radiotherapy relies on the induction of type I IFN signaling in malignant cells, tumor-infiltrating antigen presenting cells, and other immune cells within lymphoid organ or blood [38,39]. Activation of the type I IFN signature was shown to predict the efficacy of adjuvant anthracyclin-based therapies in human cancer patients [16]. Finally, animal models of human cancers suggest that type I IFN-induced expression of CXCL10 by cancer cells plays a major role in the success of anti-cancer therapies, implicating T cell-selective chemoattraction as a relevant component of this response [16]. In this context, therapeutic strategies to promote endogenous type I IFN production, or to target exogenous type I IFNs in the tumor microenvironment, are now considered effective complements in cancer therapy [17].

Our finding that MEK inhibitors trigger the molecular mechanisms of the anti-viral type I IFN-driven innate defense in human keratinocytes could contribute to the understanding of the molecular mechanisms underlying skin inflammation in patients treated with these drugs. Even more intriguing is the possibility that, through this mechanism, trametinib and cobimetinib, alone or combined with BRAF inhibitors, might sustain the activation of the anti-cancer-specific immune response, including enhanced recruitment of dendritic cells and lymphocytes due to sustained chemokine release. For instance, the promising results from clinical trials involving triple combination therapy with BRAF-MEK inhibitors and anti-PD-L1 antibodies in *BRAF*-mutated metastatic melanoma [12] may take advantage of this pro-inflammatory component. Finally, by activation of the type I IFN response in the healthy microenvironment, a combination strategy comprising MEK inhibitors could contribute to the massive recruitment of tumor-specific lymphocytes and dendritic cells and eventually improve the treatment of a variety of epithelial cancers.

## 4. Materials and Methods

### 4.1. Keratinocyte Source and Culture

This study (project identification: No. 465) was approved on 23 February 2016 by Istituto Dermopatico dell’Immacolata review board, and performed according to the Declaration of Helsinki Guidelines. Primary cultures of normal human keratinocytes were obtained from healthy donors undergoing plastic surgery (*n* = 4, two females and two males, age 25–40) as previously described [40]. Keratinocytes were grown in serum-free Keratinocyte Growth Medium (Lonza, Walkersville, MD, USA), formed of Keratinocyte Basal Medium added of EGF, hydrocortisone, bovine insulin, bovine pituitary extract, and gentamycin sulfate [15]. In the 24 h before and during the experiments, keratinocyte cultures were kept in Keratinocyte Basal Medium (Lonza).

### 4.2. Keratinocyte Lysis

RIPA buffer (20 mM Tris-HCl, pH 7.5, 150 mM NaCl, 1% Triton X-100, 1 mM EDTA, 1 mM sodium orthovanadate) supplemented with an antiprotease cocktail (Roche Diagnostics, Mannheim, Germany) was used for total cell lysis. During the whole extraction procedure and sample manipulation, cell lysates were kept on an ice bath.

### 4.3. Kinase Inhibitors

The small-molecule, cell permeant BRAF inhibitors vemurafenib and dabrafenib, and the MEK inhibitors trametinib and cobimetinib were purchased from Selleckchem (Munich, Germany). These inhibitors were dissolved in dimethylsulfoxide (DMSO). In all experiments performed on keratinocyte cultures, the DMSO concentration as vehicle control was 0.1% (*v*/*v*).

### 4.4. Western Blot Analysis

Proteins were analyzed according to our previously described protocol [15]. The primary antibodies were the following: IRF1 (#8478), phospho-Tyr701-STAT1 (#7649), phospho-ERK1/2 (#9101), ERK1/2 (#9102), IFIT1 (#12082), all from Cell Signalling Technology (Beverly, MA, USA); STAT1 (#sc-346) from Santa Cruz Biotechnology (Santa Cruz, CA, USA); IFN-κ (#H00056832-M01, clone 1B7) from Abnova GmbH (Heidelberg, Germany). Anti-actin antibody (#sc-1615, Santa Cruz Biotech.) was used for loading control of total cell lysates.

### 4.5. Quantitative Real-Time RT-PCR Analysis

Total RNA isolation and real time RT-PCR analysis were performed as previously described [15]. The primer sets were synthesized (by Sigma Aldrich, Milan, Italy) according to the following sequences: *IFN-κ* sense: GGATAGACAATTTCCTGAAAGAAAAG; *IFN-κ* antisense: TCTTGCTTGAAGGTAGATGATTCTT; *IFIT2* sense: TGGTGGCAGAAGAGGAAGAT; *IFIT2* antisense: GTAGGCTGCTCTCCAAGGAA; *CCL2* sense: AACCACAGTTCTACCCCTGGG; *CCL2* antisense: TAATGATTCTTGCAAAGACCCTCAA; *CXCL10* sense: TGGCATTCAAGGAGTACCTCTCT; *CXCL10* antisense: CTGATGCAGGTACAGCGTACG; *actin* sense: CCTCACCCTGAAGTACCCCA; *actin* antisense: TCGTCCCAGTTGGTGACGAT. Fluorescence intensity was analyzed by the ABI PRISM 5700 detection system (Applied Biosystems, Foster City, CA, USA). Determinations were produced in triplicate and the quantification was done by the comparative CT method [41].

### 4.6. Enzyme-linked Immunosorbent Assay (ELISA)

Chemokines CCL2 and CXCL10 in the culture medium were measured with dedicated kits from BD Pharmingen (San Diego, CA, USA).

### 4.7. Transfection with Specific Small Interference (si) RNA

*IRF1* and *IFN-κ* were knocked-down by using small interfering (si)RNA ON-TARGET *plus* SMARTpools (L-011704-00-0005 and L-013217-00-0005, respectively) from Dharmacon RNA Technology (Lafayette, CO, USA); a pool of four non-targeting siRNA was used as control (D-001810-10-05). Keratinocytes were transfected with *si-IRF1*, *si-IFN-κ*, or control siRNA (all at 50 nM final concentration) by using 4 μL/mL INTERFERin^®^ transfection reagent (Polyplus Transfection, Euroclone, Milan, Italy), as previously detailed [15]. After 48 h, si-RNA transfected keratinocytes were treated with the MEK inhibitors for the indicated interval and finally lysed for total RNA or protein extraction.

### 4.8. Statistical Analysis

The Wilcoxon signed-rank test (GraphPad prism Software, La Jolla, CA, USA) was applied to compare differences between groups of data. Significance was assumed at a *p*-value of 0.05 or less.

## References

[1] Samatar A.A., Poulikakos P.I. (2014). Targeting RAS-ERK Signalling in Cancer: Promises and Challenges. Nat. Rev. Drug Discov..

[2] Hanahan D., Weinberg R.A. (2011). Hallmarks of Cancer: The Next Generation. Cell.

[3] Hatzivassiliou G., Song K., Yen I., Brandhuber B.J., Anderson D.J., Alvarado R., Ludlam M.J., Stokoe D., Gloor S.L., Vigers G. (2010). RAF Inhibitors Prime Wild-Type RAF to Activate the MAPK Pathway and Enhance Growth. Nature.

[4] Poulikakos P.I., Zhang C., Bollag G., Shokat K.M., Rosen N. (2010). RAF Inhibitors Transactivate RAF Dimers and ERK Signalling in Cells with Wild-Type BRAF. Nature.

[5] Macdonald J.B., Macdonald B., Golitz L.E., LoRusso P., Sekulic A. (2015). Cutaneous Adverse Effects of Targeted Therapies. J. Am. Acad. Dermatol..

[6] Roskoski R. (2012). ERK1/2 MAP Kinases: Structure, Function, and Regulation. Pharmacol. Res..

[7] Zhao Y., Adjei A.A. (2014). The Clinical Development of MEK Inhibitors. Nat. Rev. Clin. Oncol..

[8] Dudley D.T., Pang L., Decker S.J., Bridges A.J., Saltiel A.R. (1995). A Synthetic Inhibitor of the Mitogen-Activated Protein Kinase Cascade. Proc. Natl. Acad. Sci. USA.

[9] Caunt C.J., Sale M.J., Smith P.D., Cook S.J. (2015). MEK1 and MEK2 Inhibitors and Cancer Therapy: The Long and Winding Road. Nat. Rev. Cancer.

[10] Zhao Z., Xie L., Bourne P.E. (2017). Insights into the Binding Mode of MEK Type-III Inhibitors. A Step Towards Discovering and Designing Allosteric Kinase Inhibitors Across the Human Kinome. PLoS ONE.

[11] Luke J.J., Flaherty K.T., Ribas A., Long G.V. (2017). Targeted Agents and Immunotherapies: Optimizing Outcomes in Melanoma. Nat. Rev. Clin. Oncol..

[12] Simeone E., Grimaldi A.M., Festino L., Vanella V., Palla M., Ascierto P.A. (2017). Combination Treatment of Patients with BRAF-Mutant Melanoma: A New Standard of Care. BioDrugs.

[13] Abdel-Rahman O., ElHalawi H., Ahmed H. (2015). Risk of Selected Dermatologic Toxicities in Cancer Patients Trated with MEK Inhibitors: A Comparative Systematic Review and Meta-Analysis. Future Oncol..

[14] Pastore S., Lulli D., Girolomoni G. (2014). Epidermal Growth Factor Receptor Signalling in Keratinocyte Biology: Implications for Skin Toxicity of Tyrosine Kinase Inhibitors. Arch. Toxicol..

[15] Lulli D., Carbone M.L., Pastore S. (2016). Epidermal Growth Factor Receptor Inhibitors Trigger a Type I Interferon Response in Human Skin. Oncotarget.

[16] Sistigu A., Yamazaki T., Vacchelli E., Chaba K., Enot D.P., Adam J., Vitale I., Goubar A., Baracco E.E., Remédios C. (2014). Cancer Cell-Autonomous Contribution of Type I Interferon Signaling to the Efficacy of Chemotherapy. Nat. Med..

[17] Zitvogel L., Galluzzi L., Kepp O., Smyth M.J., Kroemer G. (2015). Type I Interferons in Anticancer Immunity. Nat. Rev. Immunol..

[18] Corrales L., Matson V., Flood B., Spranger S., Gajewski T.F. (2017). Innate Immune Signaling and Regulation in Cancer Immunotherapy. Cell Res..

[19] Xu L., Zhou X., Wang W., Wang Y., Yin Y., Laan L.J., Sprengers D., Metselaar H.J., Peppelenbosch M.P., Pan Q. (2016). IFN Regulatory Factor 1 Restricts Hepatitis E Virus Replication by Activating STAT1 to Induce Antiviral IFN-Stimulated Genes. FASEB J..

[20] Komatsu Y., Derwish L., Hirasawa K. (2016). IRF1 Downregulation by Ras/MEK is Independent of Translational Control of IRF1 mRNA. PLoS ONE.

[21] Fritsche-Guenther R., Witzel F., Sieber A., Herr R., Schmidt N., Braun S., Brummer T., Sers C., Blüthgen N. (2011). Strong Negative Feedback from Erk to Raf Confers Robustness to MAPK Signalling. Mol. Syst. Biol..

[22] Zhou X., Michal J.J., Zhang L., Ding B., Lunney J.K., Liu B., Jiang Z. (2013). Interferon Induced IFIT Family Genes in Host Antiviral Defense. Int. J. Biol. Sci..

[23] Carlos G., Anforth R., Clements A., Menzies A.M., Carlino M.S., Chou S., Fernandez-Peñas P. (2015). Cutaneous Toxic Effects of BRAF Inhibitors Alone and in Combination with MEK Inhibitors for Metastatic Melanoma. JAMA Dermatol..

[24] Rauch I., Müller M., Decker T. (2013). The Regulation of Inflammation by Interferons and Their STATs. JAKSTAT.

[25] Albanesi C., Scarponi C., Bosisio D., Sozzani S., Girolomoni G. (2010). Immune Functions and Recruitment of Plasmacytoid Dendritic Cells in Psoriasis. Autoimmunity.

[26] Korl-Mäurer A., Goebeler M., Mäurer M. (2015). Cutaneous Adverse Events Associated with Interferon-Beta Treatments of Multiple Sclerosis. Int. J. Mol. Sci..

[27] López de Padilla C.M., Niewold T.B. (2016). The Type I Interferons: Basic Concepts and Clinical Relevance in Immune-Mediated Inflammatory Diseases. Gene.

[28] Delaney T.A., Morehouse C., Brohawn P.Z., Groves C., Colonna M., Yao Y., Sanjuan M., Coyle A.J. (2016). Type I IFNs Regulate Inflammation, Vasculopathy, and Fibrosis in Chronic Cutaneous Graft-Versus-Host Disease. J. Immunol..

[29] Stannard J.N., Reed T.J., Myes E., Lowe L., Sarkar M.K., Xing X., Gudjonsson J.E., Kahlenberg J.M. (2017). Lupus Skin is Primed for IL-6 Inflammatory Responses through a Keratinocyte-Mediated Autocrine Type I Interferon Loop. J. Investig. Dermatol..

[30] Wenzel J., Wörenkämper E., Freutel S., Henze S., Haller O., Bieber T., Tüting T. (2005). Enhanced Type I Interferon Signalling Promotes Th1-Biased Inflammation in Cutaneous Lupus Erythematosus. J. Pathol..

[31] Wenzel J., Bekisch B., Uerlich M., Haller O., Bieber T., Tüting T. (2005). Type I Interferon-Associated Recruitment of Cytotoxic Lymphocytes: A Common Mechanism in Regressive Melanocytic Lesions. Am. J. Clin. Pathol..

[32] Ma Y., Adjemian S., Galluzzi L., Zitvogel L., Kroemer G. (2014). Chemokines and Chemokine Receptors Required for Optimal Responses to Anticancer Chemotherapy. OncoImmunology.

[33] Lança T., Costa M.F., Gonçalves-Sousa N., Rei M., Gross A.R., Penido C., Silva-Santo B. (2013). Protective Role of the Inflammatory CCR2/CCL2 Chemokine Pathway through Recruitment of Type 1 Cytotoxic γδ T Lymphocytes to Tumor Beds. J. Immunol..

[34] Harlin H., Meng Y., Peterson A.C., Zha Y., Tretiakova M., Slingluff C., McKee M., Gajewski T.F. (2009). Chemokine Expression in Melanoma Metastases Associated with CD8+ T-Cell Recruitment. Cancer Res..

[35] Mikucki M.E., Fisher D.T., Matsuzaki J., Skizki J.J., Gaulin N.B., Muhitch J.B., Ku A.W., Frelinger J.G., Odunsi K., Gajewski T.F. (2015). Non-Redundant Requirement for CXCR3 Signalling during Tumoricidal T-Cell Trafficking across Tumour Vascular Checkpoints. Nat. Commun..

[36] Goldszmid R.S., Dzutsev A., Trinchieri G. (2014). Host Immune Response to Infection and Cancer: Unexpected Commonalities. Cell Host Microbe.

[37] Fuertes M.B., Woo S.R., Burnett B., Fu Y.X., Gajewski T.F. (2013). Type I Interferon Response and Innate Immune Sensing of Cancer. Trends Immunol..

[38] Galluzzi L., Buqué A., Kepp O., Zitvogel L., Kroemer G. (2015). Immunological Effects and Conventional Chemotherapy and Targeted Anticancer Agents. Cancer Cell.

[39] Bracci L., Sistigu A., Proietti E., Moschella F. (2017). The Added Value of Type I Interferons to Cytotoxic Treatments of Cancer. Cytokine Growth Factor Rev..

[40] Pastore S., Fanales-Belasio E., Albanesi C., Chinni L.M., Giannetti A., Girolomoni G. (1997). Granulocyte Macrophage Colony-Stimulating Factor is Overproduced by Keratinocytes in Atopic Dermatitis. Implications for Sustained Dendritic Cell Activation in the Skin. J. Clin. Investig..

[41] Pfaffl M.W. (2001). A New Mathematical Model for Relative Quantification in Real-Time RT-PCR. Nucleic Acids Res..

